# Migration and Migrant Labour in the Gig Economy: An Intervention

**DOI:** 10.1177/09500170221096581

**Published:** 2022-07-05

**Authors:** Niels van Doorn, Fabian Ferrari, Mark Graham

**Affiliations:** University of Amsterdam, The Netherlands; University of Oxford, UK; University of Oxford, UK

**Keywords:** employment regulation, gig economy, migrant labour, migration, platform economy, social policy

## Abstract

In urban gig economies around the world, platform labour is predominantly migrant labour, yet research on the intersection of the gig economy and labour migration remains scant. Our experience with two action research projects, spanning six cities on four continents, has taught us how platform work impacts the structural vulnerability of migrant workers. This leads us to two claims that should recalibrate the gig economy research agenda. First, we argue that platform labour simultaneously degrades working conditions while offering migrants much-needed opportunities to improve their livelihoods. Second, we contend that the reclassification of gig workers as employees is by itself not sufficient to counter the precarisation of migrant gig work. Instead, we need ambitious policies at the intersection of immigration, social welfare, and employment regulation that push back against the digitally mediated commodification of migrant labour worldwide.

## Introduction: Platform labour is predominantly migrant labour

On 17 April 2020, Rajesh Jayaseelan, an Uber driver who had left his wife and children in India to work in London, died alone in his apartment. Having contracted COVID-19, Rajesh spent days at home starving and too scared to call an ambulance. He was concerned about being evicted if his landlord discovered that he was infected. By the time he had been admitted to hospital it was too late to save him (Booth, 2020). Rajesh’s tragic story illustrates how migrant gig workers experience a set of compounding vulnerabilities related to their employment and residency status, exclusion from national welfare systems, unfamiliarity with local legislative frameworks, and health and safety hazards on the job.

So far, however, the study of platform-mediated gig work has not devoted much attention to the role of migrant labour and its governance at the intersection of labour market regulation, social welfare, and immigration policy (for exceptions, see Altenried et al., 2021; Barratt et al., 2020; Van Doorn and Vijay, 2021). This is surprising, given the prevalence of questions around immigration and migrant labour in key labour studies journals (e.g. Könönen, 2019; Maury, 2020). Moreover, the neglect of these questions in the literature on platform labour is a serious omission, given that migrants constitute a large and growing section of the urban gig economy workforce. While there is still little comprehensive or comparative public data on the topic and the precise number of migrant workers (and indeed workers in general) in urban gig economies remains unknown, a recent International Labour Organization (ILO) survey found that migrant workers constitute over 70 per cent of the platform-mediated delivery sectors in Argentina and Chile (ILO, 2021).

Meanwhile, there is growing evidence that migrant workers likewise provide a large share of the labour power driving gig platforms in cities ranging from New York to Paris and from Bogotá to Cape Town (Alderman, 2019; Bandeira, 2019; Dzieza, 2021). Even in India and China, countries with huge domestic labour markets, it is primarily domestic migrants^1^ who provide key gig economy services such as ride-hailing, domestic and care work, and food delivery in major cities (Chen and Qiu, 2019; Raval, 2020; Tandon and Rathi, 2021). Thus, it increasingly seems likely that, without a continuous influx of migrants, platform companies would have trouble maintaining their labour supply in many of their key markets across the globe. Accordingly, it can be argued that migrant labour serves an infrastructural role for these platforms – one as vitally important to their business model’s viability as the steady influx of investment capital.

In order to bring questions around migration to the forefront of gig economy debates, we draw on data and insights gained during two ongoing action research projects spanning six cities on four continents: Amsterdam, Bangalore, Berlin, Cape Town, Johannesburg, and New York. We have participated in and observed gig workers’ day-to-day conversations, and followed their work routines and formal and informal organising practices; we have conducted hundreds of in-depth interviews; and we have convened workshops and focus groups for workers and other stakeholders in each city. Our extensive qualitative data suggests that migration is not only highly prevalent across the particular gig economy sectors that we have studied (domestic work, food delivery, ride-hailing, and temporary staffing); it also dovetails with a range of unique labour issues that demand scholarly attention. After all, economic factors (e.g. availability of venture capital), sociocultural factors (e.g. growing urban inequality), and technological factors (e.g. app-based digital infrastructures) alone do not explain the proliferation of platform labour. Significantly more research is needed on how the public regulation of immigration and migrant labour impacts foreign workers’ opportunities and challenges in the segmented labour markets of their host countries.

In this article, we argue that there is an urgent need within gig economy scholarship to reckon with ‘the inequalities inherent in the precarisation of work deriving from capital’s constitutive relation with difference and the systematic production of heterogeneous exploitable figures’ through racialised immigration and labour market policies (Maury, 2020: 821). It is this state-sanctioned production process that shapes the conditions under which labour platforms can operate successfully, by taking advantage of exploitable migrants and their need for easily accessible work opportunities. At the same time, however, these migrants also take advantage of the opportunities labour platforms make available – even while taking on a disproportionate amount of physical, economic and mental risk.

We structure our argument around two interconnected claims that will hopefully help to recalibrate the current gig economy debate. First, while platform labour degrades working conditions, it also offers migrants much-needed income opportunities. Second, granting gig workers employee status is by itself not enough to counter the precarisation of migrant gig work, and debates about the future of the gig economy should thus not be narrowly focused on issues regarding misclassification. Instead, we argue that the exigencies of platform-mediated migrant labour require a thorough and ambitious revision of existing regulation at the intersection of immigration and employment law and welfare policy. Such a radical revision is needed to forcefully push back against the digitally mediated commodification of low-paid migrant work across the globe.

## Platform labour is simultaneously a site of degradation and opportunity

The boundaries between formal and informal work arrangements are not as sharp as they may initially seem (Chen, 2005), and labour platforms exploit this ambiguous terrain through technical and legal means. Surveying the precarious aspects of informalised labour across gig economies, Moore (2018: vi) notes that platform-based gig work is under-regulated, frequently unprotected, usually ‘does not guarantee minimum wage’, ‘does not offer income security’, ‘runs a high risk of discrimination’, and offers no occupational health standards or career-enhancing educational prospects. As such, she makes a strong case for the commonly held thesis that platform labour is degraded labour. When compared with the standard employment relationship, we can only concur. Standard employment is traditionally defined as work that is full time, indefinite, and includes statutory benefits and entitlements (ILO, 2016). Yet at the same time we wonder whether this is the most apt and ultimately the most strategically sound comparison in the light of the circumstances faced by migrant workers.

Given that the majority of workers around the world are engaged in non-standard and vulnerable forms of employment characterised by low pay and no social protection – two billion people, accounting for 61 per cent of the global workforce (ILO, 2020) – it is pivotal to ask who, exactly, experiences this kind of work as ‘degraded’. Indeed, regarding platform labour as necessarily ‘degraded labour’ risks sacrificing important questions about why particular groups of workers sign up with gig platforms in the first place. It may also focus on the question of platform culpability, at the expense of a broader structural critique of how labour market reforms and welfare retrenchment impact low-wage workers, especially women, minorities and migrants (Huws, 2020).

To begin building such a critique, we argue that labour platforms engage in *selective formalisation*: a set of business and management practices that formalise some aspects of gig work while perpetuating the precarity associated with informal labour markets (Van Doorn, 2021). Although platforms provide clients and workers with tools for documenting worked hours, evaluating service experiences, processing payments, and/or doing taxes – and despite framing the formalisation of historically informal work as a primary value proposition to both clients and policymakers – they often strategically refrain from enforcing the requirements and norms of formal employment (Ticona and Mateescu, 2018). Instead, they dissolve the formal employment relation into a nexus of non-negotiable commercial contracts and user agreements, to which nominally self-employed workers must consent if they want to retain access to the platform. These agreements tend to be opaque and are rarely offered in the languages spoken by large parts of the local workforce (Woodcock and Graham, 2019). Such practices structurally benefit platform companies and the users of the services they provide, while disempowering the workers who are expected to carry the administrative, fiscal, and legal burdens of a formal labour relation.

At the same time, as our research shows, platform labour is particularly popular among low-wage migrant workers who have traditionally been excluded from the standard employment relation. There are several reasons for this. Starting with the so-called ‘onboarding’ process itself, its low threshold and speed is frequently lauded when compared with the hiring process at other firms or temporary work agencies, which can be protracted and sometimes involves invasive interview questions or (drug) tests. The online onboarding process feels less demanding to many migrant gig workers, and for some it is how they find other (informal) gigs – via platforms such as Craigslist or eBay Kleinanzeigen. Referral bonuses make onboarding with a gig platform even more appealing, as they offer gig workers the prospect of extra income after they have completed their first number of deliveries, rides, or cleans. Moreover, whereas migrant workers regularly face various forms of discrimination and racism while looking for a job, the seemingly indiscriminate onboarding policies of most platform companies can feel like a breath of fresh air. Although such policies ultimately result in markets that are oversaturated with service providers whose work-readiness and wait times are not compensated, they initially provide a welcome opportunity for those needing to break into the labour market.

Selective formalisation also means that platform companies are often quite lax with respect to their enforcement of formal requirements such as background checks and business licences. Given that such requirements can form a significant obstacle to finding formal employment in a host country, especially in low-wage labour markets, this is a boon to migrant workers.^2^ This is especially true for undocumented migrants: until recently, food delivery companies made little effort to check who uses their accounts, giving those lacking a visa, work permit, or social security number a new income opportunity. Lately, however, companies have started to enforce their rules more rigorously, with Uber requiring workers to submit ‘live’ photos of themselves and use facial recognition to verify their account, thereby diminishing the livelihoods of this most precarious group of workers (White, 2021).

Language barriers also form a major hurdle for migrants looking for work in a foreign country, as employers frequently require applicants to speak or learn the native language – or at least speak English. Gig platforms, in contrast, offer apps that come in a variety of languages and, especially in the case of food delivery, allow workers to do their job and keep track of their earnings without much verbal communication. When it comes to these earnings, what makes gig platforms appealing to migrant workers is their use of bonus incentives during times of high demand, during which workers can make more money than they would in a minimum wage service job. Tax benefits and exemptions for the self-employed further contribute to this perceived income advantage. Moreover, many gig platforms – especially in food delivery and ride-hailing markets – give their workers the opportunity to ‘cash out’ whenever they want, instead of having to wait at least two weeks for their pay cheque.

Finally, migrant workers generally find that gig platforms grant them relatively more autonomy with respect to decisions about when (not) to work, compared with other jobs available to them (Anwar and Graham, 2022). Not only can they schedule their work hours and make last-minute adjustments when necessary, but also reject incoming offers when it is inconvenient. While this individual bargaining power may pale in comparison to collectively established formal bargaining power, it is nevertheless a form of market-based autonomy that many gig workers cherish and do not want to give up.

In sum, for those who face structural difficulties accessing secure, well-paid and properly protected jobs, gig work can present a provisional step *up*, rather than down. Yet this certainly does not mean that platform companies should be lauded. On the contrary, we should scrutinise their actions and policies according to the same standards as those used to assess other low-wage employers and labour market intermediaries. Ultimately, we want to make two points here. First, practices of selective formalisation generate deeply ambiguous arrangements in which migrants find valuable opportunities within degraded labour conditions. Second, these conditions are symptomatic of much broader structural problems at the bottom of the labour market (Doussard, 2013; Glaser, 2021). We elaborate on this second point in the next section.

## Reclassification is by itself not enough to counter the precarisation of migrant gig work

Many scholars and labour advocates consider the misclassification of platform workers as self-employed contractors to be the primary cause driving the gig economy’s widespread labour degradation and precarity. Accordingly, much critical and political energy is spent on advocating for the reclassification of gig work as a form of employment (De Stefano, 2015; Dubal, 2017). In these efforts, reclassification is frequently presented as a legislative antidote for the exploitation and predatory logics that mark platform labour. We recognise that the reclassification of platform workers as employees may provide an indispensable pathway to rights of representation by a union and recognition for collective bargaining purposes. However, we argue that such reclassification will have only limited impact if we fail to recognise that employment – as a legal and political arrangement – has frequently failed to secure the livelihoods and dignity of low-wage workers, particularly migrants and minorities. Employment, in our view, is thus a stratified site of ongoing struggle.

To be sure, this is not an argument against the hard-fought rights and protections attached to the standard employment relation. On the contrary, our point is that such rights and protections should be made available to *all workers*, regardless of their legal status. For too long, these entitlements have been eroded and unequally distributed along lines of class, race, gender, and nationality (Vosko, 2010). And where they have been granted, they have often been poorly enforced (Estlund, 2018). Indeed, these inequities have pushed some of the most vulnerable workers from precarious non-standard employment arrangements into the gig economy.

Many of the migrant gig workers we interviewed had an ambivalent perspective on the employment relation, as it formed both an aspirational object and a site of constraint and exclusion. Employment was frequently associated with limitations rather than opportunity. For example, the Dutch government caps the legal working time for students from outside the European Economic Area (EEA) at 16 hours per week, in the case of a regular employment contract. Yet it also allows these students to register themselves as self-employed contractors, in which case they can work however much they want. This legal loophole has created a massive rush among non-EEA students to join Deliveroo and Uber Eats, which came as a godsend for these companies as they were seeking to expand in key Dutch cities. Doing app-based food delivery gives these students the ability to work 30–40 hours per week during periods with low study loads, enabling them to make much more money than if they worked a minimum wage job for a maximum of 16 hours per week. At the same time, however, they take on a significant level of risk by riding around the city on e-bikes with only the limited accident insurance offered by the platforms themselves. If allowed to work more (flexible) hours, many non-EEA students said they would prefer the safety of an employment contract.

As this Dutch example illustrates, the operations and strategies of gig platforms are contingent on the intersecting regimes of immigration policy and employment regulation in different national contexts. Yet welfare regulation frequently plays a mediating role too. In Germany, for instance, immigrants from within and beyond the EU have to meet various eligibility criteria before being able to access social benefits. Even when they do gain access, Germany’s welfare-to-work reforms, commonly known as the Hartz reforms, have made such benefits contingent on the acceptance of heavily monitored and subsidised low-wage jobs. Although many of the ‘insider jobs’ in Germany’s core labour market continue to be well protected, those reforms have intensified the employment insecurity of labour market outsiders (Chih-Mei, 2018: 746). These policies have contributed to an institutional setting in which ‘low road’ employers and labour market intermediaries, including Uber and Amazon Logistics, can experiment with forms of subcontracting, exploitation and regulatory avoidance that do not rely on simple misclassification. As long as immigrants’ access to welfare services and decent work is restricted by their residency status as well as inconsistent ‘processes of bureaucratic discrimination’ (Ratzmann, 2020: 3), platform companies can promote themselves as offering a quick and accessible source of income.

To further lower entry barriers, Uber contracts with so-called Fleet Partners (i.e. private transportation intermediaries) in many urban markets, allowing the company to devolve legal responsibilities while scaling up its service. Dealing with Fleet Partners is often a less cumbersome arrangement for migrant workers, compared with the administrative work of contracting directly with Uber as self-employed drivers (Kozlowska, 2019). However, in our interviews with migrant Uber drivers we found that these subcontracted employment arrangements can also be highly opaque and precarious, with some drivers earning below minimum wage (Howson et al., 2021), and others expressing uncertainty about whether they were insured by the platform in the case of an accident.

Considering the increasing proliferation of non-standard employment relations, we argue that gig worker reclassification may be necessary, but will certainly not be sufficient. Reclassification may also inadvertently harm the most vulnerable group of migrant workers: those lacking documentation. While the illicit subleasing of courier accounts is typically marked by exploitative relations that perversely mirror the more formal Fleet Partner system, these jobs nevertheless offer a lifeline to many workers. Heightened public scrutiny has already led Uber and Deliveroo to implement stricter surveillance and enforcement techniques, but if these companies were forced to reclassify their workforce this would surely result in a purge of undocumented migrants from the platform. This is obviously not an argument against reclassification per se, but we do think that efforts to pass new regulation should be preceded by a careful consideration of ways to minimise the harm to those who have so far been excluded from employment’s protective scope. Which groups of workers is such regulation ultimately protecting and which workers will be sacrificed once it gets passed?

## Conclusion: Against the platform-mediated commodification of migrant labour

To abolish the platform-mediated commodification of migrant labour, which may ultimately entail the abolition of the gig economy as we know it, we need a radical and comprehensive policy overhaul that acknowledges how employment and welfare regulation interacts with immigration policies at a national and international level. This means we should expand our political horizon and augment gig worker reclassification efforts with struggles for broader worker protections, redistributive social policies, and immigration reforms geared toward global social justice and solidarity.

To be sure, such an overhaul will face major challenges. National governments are not merely complicit in maintaining the status quo through punitive labour and welfare reform and ‘managed migration’ policies (Menz, 2009), they have also benefitted from it. Because low-wage sectors serve the vital macro-economic function of ‘absorbing’ marginalised and unemployed workers into the formal labour market, governments have generally been reticent to substantially address the problem of degraded working conditions, including minimal enforcement of health and safety regulations. Since gig platforms serve a similarly useful absorptive function, we likewise do not expect much action from public administrations beyond piecemeal reforms. Intensive lobbying, astroturfing efforts, and regulatory capture by platform companies will only exacerbate any such reluctance. That said, we do not need to accept this situation. In our concluding section, we offer some proposals to counter the current (unsustainable) status quo.

First, there must be better representation and engagement of migrant gig workers in unions and other labour market institutions, including regulatory agencies (Heinrich et al., 2020). Workers should not only be given advice in their native language and empowered to define the themes and terms of labour struggle and negotiation, they should also play a formal role in the regulatory enforcement regimes that encompass gig work. We look here to what Però (2020) has called the ‘indie unions’ that have emerged across low-wage service industries. Perhaps the main strength of these indie unions – and the worker centres that inspired them – is that they expand the struggle against precarity and exploitation from sites of production to spaces of social reproduction, by tying the push for better labour conditions to (legal) support for immigrant communities.

Second, we need to radically rethink the integration of immigrants into labour markets, countering the deeply gendered and racialised logics currently dominating ‘managed migration’ policies. This requires a public reconsideration of how we value the reproductive work of immigrants and minorities. Rather than applauding ‘essential workers’ for their sacrifices during the COVID-19 pandemic, they need to be properly paid and protected – and clearly employment status alone has not always been able to secure these basic conditions. This speaks to a more general need to make low-wage service jobs not only better paid but also less precarious and dead-end, by ensuring ‘a stronger social safety net, public job creation, training programs, and transitional support’ where possible (Estlund, 2018: 825).

Of course, such progressive labour market and welfare policies usually remain bound by national borders and citizenship/residency status. To truly reckon with the ‘layers of vulnerability’ that disempower migrant workers as they navigate low-wage labour markets (Vosko et al., 2019), we will need to abolish the very distinction between inside and outside – moving beyond both the standard employment relationship and national citizenship. Towards this end, political and policy efforts should strive to secure *universal* labour protections, living wage standards, and access to public welfare infrastructures (including care facilities), regardless of one’s employment or legal status. Such efforts should be complemented by multilateral policy cooperation between sending and receiving countries, centred on the coordination of safe and dignified labour migration and the improvement of labour market conditions in sending countries (coupled with debt-relief or cancellation programmes).

So far, our proposals are intended to create conditions in which platform companies would no longer be able to benefit from a state-sanctioned ‘migrant division of labour’ (May et al., 2007). In other words, these proposals seek to disrupt the value chains of platforms by decommodifying labour and presenting migrant workers with better alternatives. That said, to fundamentally push back against the particular inequities of the gig economy, we also need innovative regulatory and policy measures that recognise how platform companies are *unlike* conventional ‘low-road’ employers, insofar as data capture and finance capital give them an outsized competitive advantage across industries. The pervasive datafication and financialisation of the gig economy also means that platform companies operate according to different business imperatives that move beyond conventional shareholder value logics, and essentially render these companies little more than fungible investment vehicles that can be leveraged to make money from money (Van Doorn and Badger, 2020).

As such, we need transnational regulatory arrangements for the governance of platforms, that extend beyond labour law to include consumer protection and competition legislation, while also aiming for a stricter regulation of financial markets, and elaborating on recent frameworks for the regulation of data rights, such as the General Data Protection Regulation (GDPR) (Van Dijck et al., 2019). This approach could, for instance, combine public audits of platform companies’ financial records and software systems with grassroots initiatives such as the Worker Info Exchange, which seeks to reclaim a measure of control over gig workers’ data.^3^ In this way, it connects a concerted pushback against the commodification of platform-mediated labour to a larger set of strategies that seek to counter the predatory and extractive logics driving platform capitalism, which disproportionately harm migrants and minorities.

Finally, the crucial functions that platforms perform as large employers and increasingly as logistical infrastructures, should encourage us to rethink who owns and controls them. There are promising signs from the platform cooperativism movement that aims to develop worker-owned and managed alternatives (Atkinson, 2021). However, many of these efforts will continue to face significant financial difficulties in their attempts to outcompete venture capital-backed incumbent firms, unless they receive structural government support. We can also ask why more is not being done to offer ‘public options’ that would provide alternatives for workers and customers alike, ‘forcing [corporate] platforms to take seriously the need to provide services in a different way’ (Rahman, 2018: 249). Platforms are a particularly vulnerable business model in this regard (Graham, 2020).

Ultimately, we hope that the arguments and proposals presented here will help focus debates about the gig economy on the unique positionalities of migrant workers. It is time that scholars, commentators, activists, and regulators collectively reckon with how this workforce is simultaneously made essential and disposable in urban gig economies around the world, and take serious steps to end these practices.

## Notes

1. In many cases, the domestic migrants in the gig economy speak different languages and lack access to local networks of support, making their situation not dissimilar from that of immigrant workers in other countries.

2. When gig companies do enforce certain requirements, such as having a business licence, they distribute template instructions that support and sometimes expedite the application process of their onboarded workers.

3. See https://www.workerinfoexchange.org/ (last accessed 20 October 2021).
